# Cracking the code: a head-to-head comparison of expert clinicians and artificial intelligence in diagnosing rare diseases

**DOI:** 10.1186/s13023-025-04112-5

**Published:** 2025-11-05

**Authors:** Georg Wolfgang Sendtner, Martin Muecke, Lorenz Grigull, Tim Bender, Charlotte Behning, Valentin Sebastian Schäfer

**Affiliations:** 1 Clinic of Internal Medicine III, Department of Oncology, Hematology, Rheumatology and Clinical Immunology, University Hospital Bonn, Venusberg Campus 1, 53127 Bonn, Germany; 2 Center for Rare Diseases Bonn, University Hospital Bonn, Bonn, Germany; 3 Institute of Human Genetics, University Hospital Bonn, Bonn, Germany; 4 Institute of Medical Biometry, Informatics and Epidemiology, University Hospital Bonn, Bonn, Germany

**Keywords:** Rare disease, Artificial intelligence, Diagnostic tool

## Abstract

**Background:**

Patients with rare diseases often face prolonged diagnostic journeys due to the low prevalence and diverse clinical presentations of these conditions. In Germany, specialized centers for rare diseases, established at university hospitals, offer targeted diagnostic and therapeutic care to reduce diagnostic delays. Tools like “Isabel Healthcare” can support clinicians by streamlining the differential diagnosis process and aiding in the accurate identification of rare conditions.

**Results:**

The study included 100 patients with a mean age of 44 years. “Isabel Healthcare DDx companion” and the interdisciplinary case conferences generated a total of 727 diagnosis suggestions. Among the top ten diagnoses suggested by “Isabel Healthcare DDx companion”, 28% matched at least one diagnosis identified during the interdisciplinary case conferences. The diagnoses suggested as “more likely” by “Isabel Healthcare DDx companion” showed a higher correlation with the differential diagnoses and procedures identified during the interdisciplinary case conferences, suggesting a potential alignment in clinical decision-making processes.

**Conclusion:**

This study has demonstrated the potential of the differential diagnostic tool “Isabel Healthcare DDx companion” to assist in patient diagnosis. However, discrepancies between the tool’s findings and expert decisions suggest that, although it can support clinicians in decision-making, its independent effectiveness may be limited by accurately filtering and interpreting the essential medical history required for a precise diagnosis.

## Background

A rare disease is defined as a disease that affects fewer than 1 in 2,000 people [1]. In total more than 6000 rare diseases have been found so far [2]. Affected individuals often face diagnostic delays, with an average of five years to receive the correct diagnosis [3]. The rarity of these diseases further complicates finding adequate information, the right specialists and potential treatments.

To address this issue, more than 30 centers for rare diseases have been established at university hospitals in Germany to enhance the diagnostic and therapeutic care for rare diseases [4]. These centers consist of clinicians who treat patients with rare diseases daily. Another special diagnostic instrument to help people with a suspected rare disease is the interdisciplinary case conference. In this conference different specialized clinicians come together to facilitate faster diagnosis of rare diseases [5, 6].

In the last years artificial intelligence (AI) has made significant advancements in the medical field [7]. Image interpretations, particularly at the department for radiology or pathology, have increasingly become a focus of interest. However, only a few AI models have been successfully implemented in clinical practice, likely due to their error- prone nature, complexity and slow processing rates [8]. Furthermore, differential diagnostic tools such as “Isabel Healthcare DDx companion” have been developed to aid and support clinicians in finding the correct diagnosis for patients. Not only do these tools integrate symptoms or clinical findings, but they can also include laboratory markers into the diagnostic process. So far, images of dermatological manifestations, for example, cannot be included. The differential diagnostic tool “Isabel Healthcare DDx companion” was first developed in 1999 by a charitable organization following the tragic death of three-year-old Isabel Maude [9]. Initially designed for acute pediatric cases, the tool was subsequently expanded to include adult conditions in 2006. This diagnostic aid has been validated through various studies involving both pediatric and adult patients, demonstrating a high level of diagnostic accuracy [10–14]. The diagnosis suggestions provided by “Isabel Healthcare DDx Companion” are further ranked as “more likely”, “likely”, and “less likely”, based on the probability of the correct diagnosis. Even though this tool has been especially validated in acute medicine, its approach could be also beneficial for patients with suspected rare diseases. Several studies have been conducted to demonstrate the specificity and impact of such differential diagnostic tools in clinical practice [15–18]. However, these studies often employed a retrospective design, which may introduce bias into the input for the diagnostic tool. In this study we used a prospective design to assess whether a differential diagnostic tool can accurately filter medical input to find the same diagnoses that match the diagnoses agreed upon by experts involved in the interdisciplinary case conferences of the center for rare diseases.

## Methods

This monocentric prospective observational study was conducted at the center for rare diseases at the University Hospital of Bonn, Germany. All patients provided written consent to participate and had their data analyzed by “Isabel Healthcare DDx companion” before being included in this study. Data was analyzed anonymously. Inclusion criteria were patients between 18 and 65 years old with an undiagnosed condition persisting for at least six months and suspicion of a rare disease expressed by a treating physician or the patients themselves. Additionally, patients needed to be physically and mentally capable of answering questions and providing written medical histories.

Between October 2020 and January 2022, 100 participants aged 18 to 64 were planned for inclusion in this study. All the cases were discussed first in interdisciplinary case conferences comprising, among others, the disciplines of general medicine, rheumatology, orthopedics, neurology, psychosomatic medicine and psychotherapy and human genetics. The conference received the same patient information as provided to “Isabel Healthcare DDx companion”. The participants were surveyed via a questionnaire regarding the following characteristics, before the case was discussed at the interdisciplinary case conference: Age, gender (m/f/non-binary), size, weight, symptoms, clinical signs, previous medical conditions, current therapies, previous therapies and laboratory parameters. The interdisciplinary case conferences excluded diagnoses, established differential diagnoses, and made decisions regarding further procedures. Unless otherwise specified, differential diagnoses and decisions regarding further procedures are compared collectively against “Isabel Healthcare DDx companion”. The web- based “Isabel Healthcare DDx companion” was used for analysis and will be referred to as “Isabel Healthcare” in the following sections. The evaluation of patient data using “Isabel Healthcare” occurred after the interdisciplinary case conferences. It did not influence treatment, therapy, or diagnoses made by the center for rare diseases. At the time of data processing by “Isabel Healthcare”, the differential diagnostic tool encompassed 9552 possible categories of suggestions (including 5946 disease diagnoses and 3606 drugs).

Suspected diagnoses generated by “Isabel Healthcare” were not disclosed to patients or members of the interdisciplinary case conferences. All patients received treatment from the center for rare diseases independent of this study, and diagnostic and therapeutic recommendations were unaffected by the software. There is no financial collaboration with the software manufacturer of “Isabel Healthcare”.

This study was approved by the Ethics committee of the University of Bonn (Lfd. Nr. 019/20).

### Statistical analysis

Statistical analyses were performed using R version 4.3.0 [19]. The number of diagnoses is reported as median and interquartile range (IQR). Absolute and relative frequencies of matching diagnoses between clinicians and “Isabel Healthcare” were calculated. The top ten diagnosis suggestions generated by “Isabel Healthcare” were categorized as “more likely”, “likely” and “less likely”, according to the manufacturer’s specifications. The number of these subgroups was assessed within the top ten diagnosis suggestions. The clinical diagnoses made by the interdisciplinary case conferences were further divided into suspected diagnosis, differential diagnosis and decisions regarding further procedures.

## Results

### Epidemiological insights and clinical profiles

**Fig. 1:**
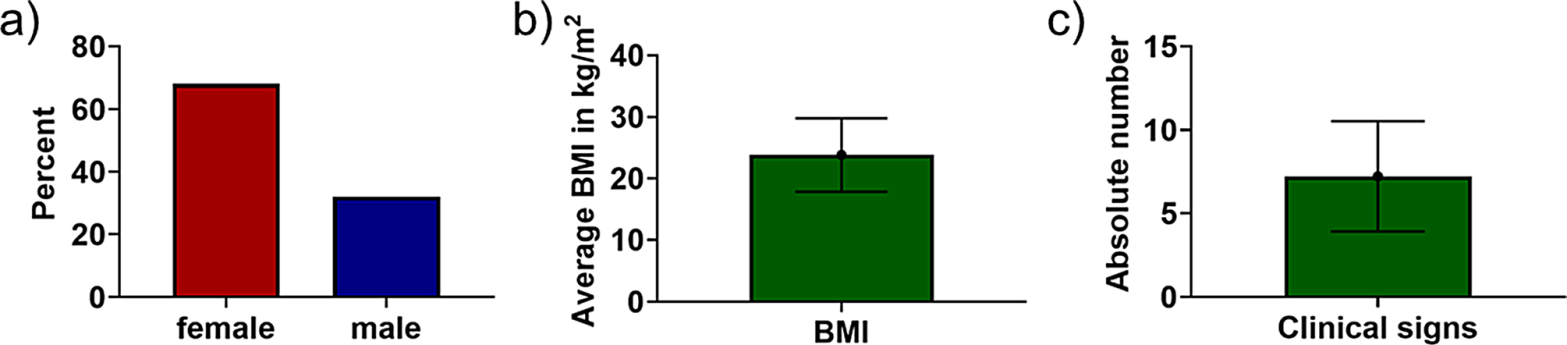
Epidemiological information. Epidemiological information of study cohort with n=100 patients. (**A**) Gender distribution of this study. No patients identified as non-binary. (**B**) Body mass index (BMI) in kg/m^2^. (**C**) Mean number of clinical symptoms. Error bar indicates standard deviation

To study the role of the differential diagnostic tool “Isabel Healthcare”, we conducted a prospective study including 100 patients. Of these, 32% were male and 68% were female. None of them identified as non-binary (Fig. 1a). The mean age was 44 (range 18–64), and the mean BMI was 23.8 (SD 5.98) (Fig. 1b). The mean number of clinical symptoms per patient was 7 (Fig. 1c), whereas the mean number of clinical signs was low (< 1). Several patients had a substantial number of prior diagnoses, underscoring the complexity of their medical histories (Table 1). The median number of previous diagnoses was two with a range from zero to eleven (Table 1). Notably, one patient had a previously identified prothrombin mutation and another had a COL2A1 mutation. Allergies and currently used medications were also recorded, providing a comprehensive overview of the patients’ health status and potential factors influencing diagnostic outcomes (Table 1). The term “case” is defined in the following text as an instance of a patient. There are a total of 100 unique cases.

### Comparison of the diagnosis suggestions made by “Isabel Healthcare” to those of the interdisciplinary case conferences

**Fig. 2:**
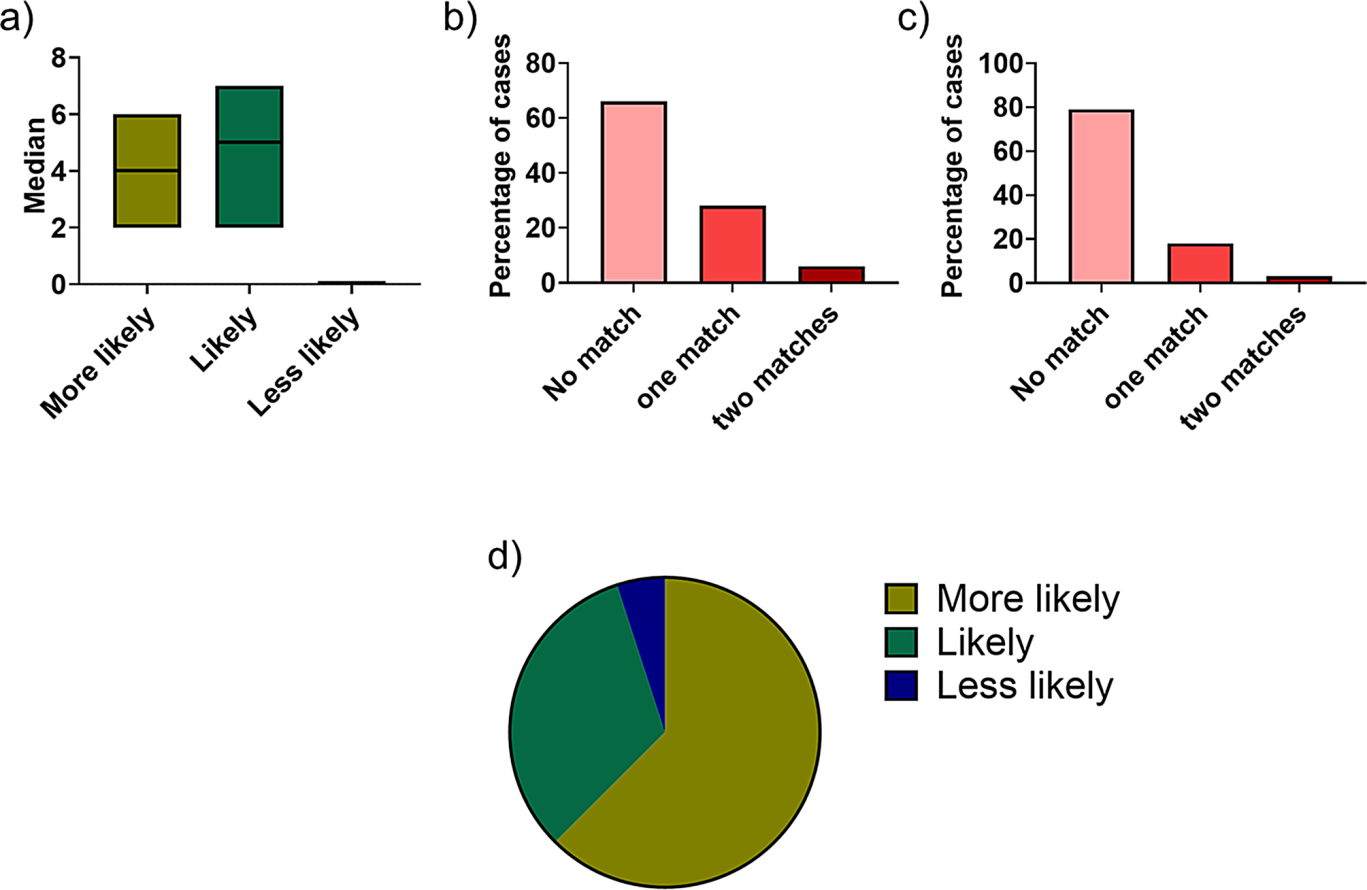
Matching suggested diagnoses between interdisciplinary case conferences and “Isabel Healthcare” (**A**) Median with 25% quantile and 75% quantile showing diagnosis suggestions by “Isabel Healthcare”. Only the first ten suggested diagnoses were included (**B**) Percentage of cases in which the diagnoses and further procedures suggested by the interdisciplinary case conferences matched those of “Isabell Healthcare”. Only the top ten suggested diagnoses. made by “Isabel Healthcare” were compared, independent from their ranking. (**C**) Percentage of cases in which only the further procedures suggested by the interdisciplinary case conferences matched those of “Isabel Healthcare”. Only the top ten suggested diagnoses ranked by “Isabel Healthcare” were compared. (**D**) Pie chart illustrating the percentage of matching diagnoses between the interdisciplinary case conferences and “Isabel Healthcare”, categorized into the levels “more likely”, (n = 25), “likely”, (n = 13), and “less likely”, (n = 2). Only the top ten suggested diagnoses ranked by “Isabel Healthcare” were compared. In total, 40 diagnoses identified by the interdisciplinary case conferences matched the top ten suggested diagnoses generated by “Isabel Healthcare”

The “Isabel Healthcare” tool grades its diagnostic suggestions into three categories: “more likely”, “likely” and “less likely”. In all cases more than ten diagnosis suggestions were made per case by “Isabel Healthcare”. As “Isabel Healthcare” presents the most likely diagnosis suggestions in the beginning, we decided to split the output into the top ten diagnosis suggestions and those ranked 11 or higher. Among the top ten diagnosis suggestions, the median number of “more likely” diagnosis suggestions was 4 (25% quantile 2, 75% quantile 6), whereas the median number of “likely” diagnosis suggestions was 5 (25% quantile 3, 75% quantile 7) (Fig. 2a). The median number of diagnosis suggestions ranked as “less likely” was zero, indicating only a small number for this subclass among the first ten diagnosis suggestions by “Isabel Healthcare” (Fig. 2a).

The total number of unique diagnoses suggested by the interdisciplinary case conferences and “Isabel Healthcare” together was 727. The interdisciplinary case conferences alone identified 253 suspected diagnoses (median per patient: 2, mean per patient: 2.53) and made 129 recommendations for further procedures (Table 2). A total of 4016 diagnosis suggestions were generated by the “Isabel Healthcare” system for the 100 cases. (Table 2). For 66% of the participants, none of the diagnosis suggestions made by the interdisciplinary case conferences were present in the top ten diagnoses suggested by “Isabel Healthcare”. For 28% of the participants, one diagnosis suggestion matched, and for 6% of participants, two diagnosis suggestions matched the diagnoses made by interdisciplinary case conferences (Fig. 2b).

If we consider only the recommendations regarding further procedures made by the interdisciplinary case conferences, in 18% of cases, one recommendation matched the top ten recommendations made by “Isabel Healthcare” and in 3% of cases, even two recommendations matched the top ten diagnosis suggestions (Fig. 2c).

Next, we aimed to investigate how the categories of “more likely,” “likely,” and “less likely” diagnoses made by “Isabel Healthcare”, have reflections on the frequency of achieving matches with the diagnoses of the interdisciplinary case conferences. This analysis helps to determine whether higher-ranked diagnoses from “Isabel Healthcare” match more frequently with those from the interdisciplinary case conferences compared to lower-ranked diagnoses.

Among the 432 diagnosis suggestions graded as “more likely”, 25 diagnoses also appeared in the possible diagnoses of the interdisciplinary case conferences (Fig. 2d). For the “likely” category, a total of 484 were made by “Isabel Healthcare”, but only 13 appeared in the diagnoses made by interdisciplinary case conferences (Fig. 2d). For the “less likely” category, a total of 84 diagnosis suggestions were made by “Isabel Healthcare”, but only two matched the possible diagnoses of the interdisciplinary case conferences (Fig. 2d). Based on these results, it appears that the most frequently matching diagnoses are indeed found within the “more likely” category generated by “Isabel Healthcare”.

## Discussion

### Differential diagnostic tool requires clinician input for accuracy

In this study, we investigated whether a differential diagnostic tool could effectively filter non-adapted medical information in a similar manner to an interdisciplinary case conference, using a prospective design. We figured out that the suggested diagnoses from the interdisciplinary case conferences and “Isabel Healthcare” sufficiently matched, particularly for high-ranked suggestions (Fig. 2b and d). This may suggest that the tool may have utility in clinical settings, especially when prioritizing likely diagnoses.

However, many diagnoses did not match, highlighting a key limitation of this particular differential diagnostic tool: its performance may be compromised when operating solely on unfiltered, non-adapted medical information. This discrepancy suggests that these tools may struggle to accurately process raw clinical data, which often contain a wide range of symptoms and medical history details that may not be directly relevant to the specific diagnosis. Without the context and filtering provided by a clinician, the tool may generate a broader or less accurate list of potential diagnoses, leading to reduced effectiveness. This issue is further supported by a multicenter, randomized study, which demonstrated that the timing of using a differential diagnostic tool influences both the number of differential diagnoses and the accuracy of the final diagnosis [20]. This limitation is not exclusive to the differential diagnosis system “Isabel Healthcare”, but has also been observed in the limited accuracy of various online symptom checkers [21, 22]. However, the differential diagnosis tool can assist clinicians in broadening their consideration of potential differential diagnoses, which can then be systematically excluded. There is evidence, that a differential diagnoses list containing the correct diagnosis can support clinicians in decision-making and reduce diagnostic errors [23].

In summary, “Isabel Healthcare” only partially provided a diagnosis similar to that of the interdisciplinary case conferences in this study. Combining these results with findings from other studies, this suggests that the diagnostic accuracy of differential diagnostic tools could be improved when used in conjunction with expert clinical judgment rather than as standalone solutions. Furthermore, it implies that, while differential diagnostic tools can assist clinicians in identifying the correct diagnosis, they may also help to reduce the rate of false diagnoses.

In contrast to our prospective study design, several retrospective studies have shown increased diagnostic accuracy when differential diagnostic tools are provided with biased data from case reports or information that has been pre-filtered and pre-graded by a clinician [10, 12, 24, 25]. Large language models, such as Bard and ChatGPT, have been shown to outperform human consensus in retrospective analyses involving publicly available complex and rare cases [26]. The performance of ChatGPT in rare disease diagnosis can be further enhanced by incorporating adaptive retrieval mechanisms, which allow the model to access and integrate relevant clinical information more effectively [27]. Recent literature also describes a new diagnostic support system for rare diseases that has outperformed even ChatGPT. This system operates in a phenotype-orientated question-and-answer format, which holds potential for enhancing clinical practice. However, it is crucial to note that the evaluation of this new system, like many studies on diagnostic tools, was conducted using retrospective data [28]. While retrospective analyses provide valuable insights, they do not fully replicate the dynamic and often unpredictable nature of real-time clinical decision-making. Therefore, a gap remains in the literature regarding the prospective validation of these tools in live clinical settings.

Even though differential diagnostic tools may improve clinical outcomes, the practical use appears to be limited to complex cases. It has been demonstrated, that the time required to use these tools and integrate them into the workflow can lead to low adoption rates in primary care settings, as observed in the UK [29].

### Limitations

This study has several critical limitations. First, follow-up restrictions due to data regulations prevented us from knowing if definitive diagnoses were later confirmed; therefore, the diagnoses of the interdisciplinary case conferences were used as the “gold standard.” A retrospective study design could have mitigated this, but it was not the focus of this investigation. Nonetheless, retrospective data can introduce bias due to case pre-selection, potentially obscuring symptoms unrelated to the selected disease, which limits the ability to replicate the clinician’s real-world diagnostic process. Second, “Isabel Healthcare” is a differential diagnostic tool designed to generate a broad range of diagnoses, even those less likely. This study found that its “more likely” diagnosis suggestions often matched the interdisciplinary case conferences outcomes (Fig. 2b and d). It would be valuable to explore whether graded input from the interdisciplinary case conferences could enhance the tool’s diagnostic accuracy. Despite these limitations, this study adds value to the literature with its prospective design. It underscores the limitations of differential diagnostic tools in real-world practice while highlighting their potential to complement clinical decision-making and improve patient safety.

## Conclusion

In this study, we showed that the differential diagnostic tool “Isabel Healthcare” can assist in identifying patient diagnoses. However, discrepancies between the tool’s output and the interdisciplinary case conferences were observed, indicating that while “Isabel Healthcare” can aid clinicians when filtered input is applied, it may not yet be fully effective on its own. Our findings highlight the potential of tools like “Isabel Healthcare” in the diagnostic process but emphasize the essential role of clinicians in filtering and contextualizing medical information.

**Table 1:** Patient characteristics

Patient characteristics	
Age	Range 18–64, mean 44
BMI	23.8 kg/m^2^, SD 5,98
Clinical symptoms	Range 0–18, mean 7.22, median 7
Clinical signs	Range 0–4, mean < 1
Previous diagnoses	Range 0–11, mean 2.76, median 2
Abnormal lab results	Range 0–8, median 0.5
Allergies	Range 0–8, mean 1
History of pregnancy	68/68 female
Number of drugs taken	Range 0–16, mean 4.1, median 4

Table describing the characteristics of patients at the time of inclusion in this study. Note that all females included in this study had a history of pregnancy. SD = Standard deviation, BMI = Body mass index

**Table 2:** Characteristics of suggested recommendations being made by interdisciplinary case conferences and “Isabel Healthcare”

	Interdisciplinary case conferences		“Isabel Healthcare“
Suggested recommendations	Suggested differential diagnoses	Recommendations regarding further procedures	Suggested differential diagnoses
Total	253	129	4016
Median	2	1	41
Mean	2.53	1.29	34.98

Please note that the total number of diagnoses represents the cumulative count of all listed diagnosis suggestions, rather than the number of distinct coded diagnoses. The interdisciplinary case conferences established differential diagnoses and made decisions regarding further procedures. In contrast, "Isabell Healthcare" only suggests differential diagnoses, but does not provide recommendations regarding further procedures

## Data Availability

The datasets used and/or analyzed during the current study are available from the corresponding author on reasonable request.
